# Digital algorithm-guided insulin therapy in home healthcare for elderly persons with type 2 diabetes: A proof-of-concept study

**DOI:** 10.3389/fcdhc.2022.986672

**Published:** 2022-09-23

**Authors:** Julia Kopanz, Julia K. Mader, Klaus Donsa, Angela Libiseller, Felix Aberer, Marlene Pandis, Johanna Reinisch-Gratzer, Gisela C. Ambrosch, Bettina Lackner, Thomas Truskaller, Frank Michael Sinner, Thomas R. Pieber, Katharina M. Lichtenegger

**Affiliations:** ^1^ Division of Endocrinology and Diabetology, Department of Internal Medicine, Medical University of Graz, Graz, Austria; ^2^ JOANNEUM RESEARCH Forschungsgesellschaft mbH, HEALTH, Institute for Biomedicine and Health Sciences, Graz, Austria; ^3^ AUSTRIAN RED CROSS, Graz, Austria

**Keywords:** digital, algorithm, insulin therapy, home healthcare, elderly, type 2 diabetes

## Abstract

GlucoTab@MobileCare, a digital workflow and decision support system with integrated basal and basal-plus insulin algorithm was investigated for user acceptance, safety and efficacy in persons with type 2 diabetes receiving home health care by nurses. During a three months study nine participants (five female, age 77 ± 10 years, HbA1c 60 ± 13 mmol/mol (study start) vs. 57 ± 12 mmol/mol (study end) received basal or basal-plus insulin therapy as suggested by the digital system. In total 95% of all suggested tasks (blood glucose (BG) measurements, insulin dose calculations, insulin injections) were performed according to the digital system. Mean morning BG was 171 ± 68 mg/dL in the first study month vs. 145 ± 35 mg/dL in the last study month, indicating a reduced glycemic variability of 33 mg/dL (standard deviation). No hypoglycemic episode < 54 mg/dL occurred. User’s adherence was high and the digital system supported a safe and effective treatment. Larger scale studies are needed to confirm findings under routine care.

**German Clinical Trials Register ID:**

DRKS00015059

## Introduction

Type 2 diabetes is a complex disease in elderly persons and persons with type 2 diabetes show higher mortality, are more likely to get institutionalized, have greater risks for multiple comorbidities (1, 2), such as heart failure, coronary artery disease, renal and liver disease, dementia, and increasing risk of hypoglycaemia (3). Elderly persons with type 2 diabetes are more affected by several geriatric syndromes (1, 2). To maximize the quality of life for elderly persons with type 2 diabetes, diabetes management and an optimal treatment of the disease must take into account not only medical, but also psychological, functional, and social geriatric aspects (2).

Health care systems and healthcare professionals are challenged to provide high quality diabetes care for elderly persons with type 2 diabetes (4, 5). Special attention should be paid to home healthcare which supports people to improve their functional status, to promote quality of life, and to prevent institutionalization (6).

In elderly persons with type 2 diabetes basal insulin is recommended to simplify diabetes management, as basal insulin is easy to handle (e.g. pre-filled pen, injection only once daily) and has a low risk of inducing hypoglycemia (1–5, 7, 8). This management simplification can have an important positive impact on public health, as hospitalizations and emergency department visits can be reduced. In addition, it may contribute to maintaining the person´s overall health status (9).

Clinical decision support systems and evidence-based algorithms have been designed to facilitate optimized glycemic control while reducing the occurrence of hypoglycemic events, and they were implemented in a digital tool which has been specifically developed to be used by non-diabetes specialists and nursing staff (10–16). An algorithm for basal and basal-plus insulin therapy that is adjusted according to the person’s individual health status has been developed for home healthcare and incorporated into a digital workflow and decision support system (GlucoTab@MobileCare). This study investigated user acceptance, safety, and efficacy of the digital algorithm-guided system for glycemic management in persons with type 2 diabetes who received home healthcare.

## Methods

### Study design

This open-label, single-centre, non-controlled proof-of-concept study was approved by the ethical board of the Medical University of Graz (EK.-No. 30-287 ex 17/18) and the legal authorities, and was performed according to the principles of Good Clinical Practice in accordance with the Declaration of Helsinki.

The study was conducted in two home healthcare sites of the AUSTRIAN RED CROSS Graz, a regional, private, non-profit organization. Persons with type 2 diabetes aged ≥ 18 years, who were treated with insulin therapy and received home healthcare by nurses of the AUSTRIAN RED CROSS Graz were included. Main exclusion criteria were any disease or condition which the investigator considered to interfere with the study or safety of the person, any mental condition rendering the person incapable of giving consent, or terminally ill persons. Before obtaining written informed consent, all participants received oral and written study information.

The study included a total of nine participants: five female, age 77 ± 10 years, care dependency: 22% level 1 (nursing care need: more than 65 hours/month), 22% level 2 (nursing care need: more than 95 hours/month), 33% level 3 (nursing care need: more than 120 hours/month), 22% level 4 (nursing care need: more than 160 - 180 hours/month), BMI 28 ± 5 kg/m^2^, glycemic control according to health status (defined by physician taking into account certain parameters, e.g. number of comorbidities, care dependency, etc.) (4): 11% good (fasting blood glucose (FBG) target 90 - 130 mg/dL), 67% moderate (FBG target 90 - 150 mg/dL), 22% frail (FBG target 100 - 180 mg/dL). Insulin dose calculation was performed following the digital systems’ suggestions under supervision of the AUSTRIAN RED CROSS healthcare nurses. The basal insulin dose was reduced by two units if any BG value was < 20 mg/dL below target and by 4 units if any BG value was > 20 mg/dL below target. The basal insulin dose was increased according to a titration table. If participants also received basal-plus insulin therapy, the bolus insulin component was estimated initially with 20% of the basal insulin dose. If necessary, investigators were contacted and reviewed the suggestion for correctness and plausibility (17).

Participants received basal or basal-plus insulin therapy (insulin glargine U300, Toujeo^®^ Solostar^®^, insulin glulisine Apidra^®^ Solostar^®^) once daily as suggested by the algorithm-guided digital system during the three months study period. BG corrections were performed with short acting insulin for very high BG values (tight/moderate glycemic control ≥ 300 mg/dL; frail glycemic control ≥ 350/dL). Eight participants completed the study according to study protocol, one participant withdrew due to hospitalization (unrelated to study) but data was included for analysis as the participant had fulfilled the treatment duration.

Additionally, nurses who were working with the digital system were asked to complete a questionnaire to assess user acceptance before study-start and at study-end on a six-point Likert scale. Participants and their relatives were asked to complete a questionnaire to assess satisfaction with the digital system at study-end on a six-point Likert scale.

### Digital system

The digital system is a telemedical point-of-care solution for diabetes management including decision support for automated suggestions for insulin dose, adjustments regarding basal insulin therapy and changes of the insulin-regimen. Suggestions for BG measurement frequency, workflow support by visualization of open tasks, and support with a documentation interface are provided for healthcare professionals. In this study the digital system was used by nurses in home healthcare and by physicians employed at a diabetes outpatient clinic after a study-related group training before use.

The basal insulin algorithm incorporated in the digital system supports healthcare professionals with the decision on which basal insulin dose to start with, when to adjust basal insulin dose, how much correctional insulin to administer if very high BG values occur, and it recommends an adequate BG measurement frequency according to health status and to previous BG levels. The algorithm has been incorporated in the GlucoTab^®^ software to reduce user workload and increase safety by preventing users from having to calculate doses etc. themselves. This basal insulin algorithm is assumed to be safer, more flexible and less complex than standard diabetes care (4, 8, 18).

The digital system is a client-server software system that consists of a mobile, tablet-based client and a backend server. The client acts as a user interface and communicates with the server *via* a mobile network.

The system provides two main functionalities for healthcare professionals:

First: Support in managing the treatment workflow for persons with type 2 diabetes who require home healthcare by providing a) automated workflow support which includes reminders for open tasks, b) facilitated ordering of glucose-lowering medication and documentation of medication administration as well as other parameters relevant for diabetes c) and visualization of BG, medication and nutrition.

Second: Support in performing the subcutaneous basal insulin therapy, especially tailored to participants, by providing a) insulin dose recommendations for an initial basal insulin dose and basal insulin titration support based on previous BG values and insulin doses. An authorized healthcare professional must confirm the suggested insulin dose in regular intervals, b) workflow support for varying intensity of BG measurements (full daily profile during therapy initialisation phase versus fasting values only for ongoing therapy), c) workflow support and dose calculation for basal-plus therapy which means administration of a short-acting insulin together with a defined meal (17).

### Data management und statistical analysis

Data were recorded in the digital system, source data form, and on electronic case report forms in the application OpenClinica^®^ (GNU, USA). Statistical analysis was conducted on intention-to-treat basis and numerical data, means/standard deviations, and medians/interquartile ranges were estimated. Categorical variables were calculated as counts and percentages. Data analysis was performed using SAS 9.4. (SAS Institute Inc., USA) as well as R Statistics 3.1.2 (GNU, Austria). For questionnaires analysis the software LimeSurvey (GNU, Germany) was used.

## Results

### User acceptance

In total 95% of all suggested tasks (BG measurements, insulin dose calculations, insulin injections) were performed according to the digital system.

The suggested calculated initial basal insulin dose was accepted for one participant (11.1%) and changed for eight participants by the treating physician (88.9%). Adherence to the suggestions of the digital system was very high: A total of 94.9% of all suggested BG measurement frequencies, 95.9% of all suggested basal insulin titration doses, 99.7% of all suggested basal insulin injection doses, 97.9% of all suggested bolus insulin injection doses and 100% of all suggested time points of titrations were accepted and performed by healthcare professionals.

All nurses (n = 9) and all participants/relatives (n = 9) consistently reported positive user satisfaction (questionnaire results: Appendix). After the end of the study all participants/relatives reported that the BG control had improved. Nurses’ perception increased post-study: By using the basal insulin algorithm, the individual BG adjustment of the participants was performed more efficiently, errors and acute diabetes- related hospitalizations could be avoided.

### Safety and efficacy

**Figure 1:**
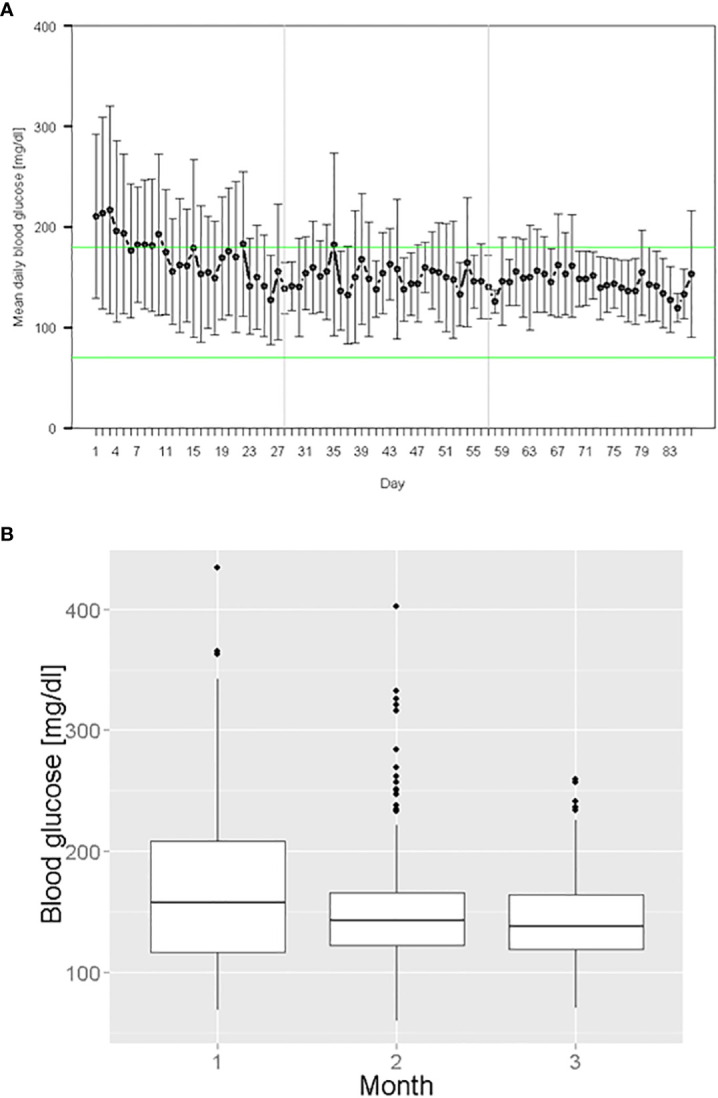
Glycemic management during digital algorithm-guided insulin therapy: **(A)** Mean daily morning BG over all treatment days **(B)** Morning BG values grouped by study month.

Including all treatment days, the mean morning BG was155 ± 53 mg/dL (n = 761) and the mean total BG was 167 ± 62 mg/dL (n = 994) averaging all participants per month. Mean morning BG decreased from 171 ± 68 mg/dL in the first study month, to 150 ± 46 mg/dL in the second study month and to 145 ± 35 mg/dL in the last study month. The glycemic variability (standard deviation) decreased by 33 mg/dL from first study month to last study month (**Figure 1**). HbA1c decreased from 60 ± 13 mmol/mol (study start) to 57 ± 12 mmol/mol (study end). No hypoglycemic episode < 54 mg/dL occurred. Morning BG values in the ranges < 70 mg/dL, 70 - 180 mg/dL, > 180 mg/dL and > 300 mg/dL were present in 0.3%, 77.0%, 22.7% and 2.6% of the blood glucose measurements, respectively. The daily mean initial insulin dose was 24 ± 13 IU and the daily mean insulin dose on the last treatment day was 38 ± 31 IU. The amount of injected total daily insulin ranged from 2 to 107 IU.

Adjustments of the insulin dose were regularly indicated by the digital system. On average the amount of insulin was adjusted nine times per participant in the study period. Adjustments were more frequent in the first four weeks (n = 44) as compared to the following weeks (week 5 - 12) (n = 41).

Seven adverse events (AEs) occurred, of which one was a serious adverse event (SAE) (hospitalization due to dyspnea, cough, and pressure on the chest; death). The SAE was not study drug- or device-related and the ethical committee and legal authorities were informed about the SAE. For one AE a causality with the study drug was probable (hypoglycemia: 58 mg/dL) while it was unlikely for all other AEs. Causality with the medical device was unlikely for all AEs.

## Discussion

The digital system supported an effective and safe treatment in participants with type 2 diabetes tested in the home healthcare setting for the first time. The use of basal insulin as a simple therapy with low risk of hypoglycemia combined with the recommended use of a clinical decision support tool in diabetes care (19) led to no severe hypoglycemic event and through continuous automated adjustments of insulin doses to stable insulin therapies with reduced glycemic variability and high adherence. Similar results concerning the efficacy, safety and adherence of suggested insulin doses were proven with GlucoTab in the hospital setting (16, 20).

Our data demonstrated, that the algorithm works with a few post-prandial BG values caused by the fact that patients sometimes had already eaten breakfast at the time nurses arrived. This is an indicator for the robustness of the algorithm concept and implicates a differentiation between pre- and post-prandial BG values in future versions of the digital system. The informative value of the results is limited by a small sample size. Thus, larger scale future studies are needed to confirm the findings regarding safety and efficacy in a more heterogeneous study population in routine use by general practitioners.

In the long term the digital system could improve quality and safety for basal insulin therapy in persons with type 2 diabetes at home.

## Data availability statement

The datasets presented in this article are not readily available as ownership belongs to JOANNEUM RESEARCH and Medical University of Graz. Requests to access the datasets should be directed to franz.feichtner@joanneum.at.

## Ethics statement

The studies involving human participants were reviewed and approved by Medical University of Graz. The patients/participants provided their written informed consent to participate in this study.

## Author contributions

JK: Conzeptualization, methodology, visualization, writing - original draft. JM: Conzeptualization, methodology, investigation, writing – review & editing. KD: Conzeptualization, methodology, software, project coordination, formal analysis, writing – review & editing, visualization. AL: Conzeptualization, methodology, project administration, writing - review & editing. FA: Investigation, writing – review & editing. MP: Investigation, writing – review & editing. JR-G: Resources, writing – review & editing. GA: Project administration, writing – review & editing. BL: Data curation, formal analysis, validation, writing – review & editing. TT: Software, data curation, writing – review & editing. FS: Funding acquisition, writing – review & editing. TP: Funding acquisition, writing – review & editing, supervision. KL: Funding acquisition, conzeptualization, methodology, writing – review & editing. All authors contributed to the article and approved the submitted version.

## Funding

Zukunftsfonds Steiermark (Projekt ABT08-183051/2016-22).

## Acknowledgments

The authors thank all the collaborating healthcare professionals from the AUSTRIAN RED CROSS Graz for their cooperation and effort. Further the authors especially acknowledge the critical review of the manuscript and the editorial assistance of Selma Mautner. The guarantor`s name taking responsibility for the content of this article is Thomas Pieber. References to prior publication of results of this study in abstract form are the 12th International Conference on Advanced Technologies & Treatments for Diabetes (2019), the 14. Gemeinsamer Österreichisch-Deutscher Geriatriekongress and the 59. Kongress der Österreichischen Gesellschaft für Geriatrie und Gerontologie (2019), the 47. ÖDG-Jahrestagung (2019), the Netzwerk Altersmedizin Steiermark Jahreskongress (2019), the 13th International Conference on Advanced Technologies & Treatments for Diabetes (2020), the 48. ÖDG-Jahrestagung (2020), the NKG 25 Nordic Gerontology Congress (2021), the 14th International Conference on Advanced Technologies & Treatments for Diabetes (2021), 81st Scientific Sessions American Diabetes Association (2021).

## Conflict of interest

JM, FS, TP and KD are founders of the decide Clinical Software Ltd. JM is a member in the advisory board of Boehringer Ingelheim, Eli Lilly, Medtronic, Prediktor A/S, Roche Diabetes Care, Sanofi-Aventis and received speaker honoraria from Abbott Diabetes Care, AstraZeneca, Dexcom, Eli Lilly, MSD, NovoNordisk A/S, Roche Diabetes Care, Sanofi, and Servier. FA received speaker honoraria from Eli Lilly, Merck Sharp & Dome, Boehringer Ingelheim, Astra Zeneca, Sanofi Aventis, Amgen and travel grants from Sanofi, Novo Nordisk, Takeda, Merck Sharp & Dome and Amgen. TP is an advisory board member of Novo Nordisk A/S, consultant for Roche Diabetes Care, Novo Nordisk A/S, Eli Lilly & Co, Infineon, Carnegie Bank, shareholder of decide Clinical Software GmbH, and is on speaker’s bureau of Novo Nordisk A/S and Astra Zeneca.

The remaining authors declare that the research was conducted in the absence of any commercial or financial relationships that could be construed as a potential conflict of interest.

## Publisher’s note

All claims expressed in this article are solely those of the authors and do not necessarily represent those of their affiliated organizations, or those of the publisher, the editors and the reviewers. Any product that may be evaluated in this article, or claim that may be made by its manufacturer, is not guaranteed or endorsed by the publisher.

## Abbreviations

AE, adverse event; BG, blood glucose; FBG, fasting blood glucose; SAE, serious adverse event.
