# Supplementary material for: Digital algorithm-guided insulin therapy in home healthcare for elderly persons with type 2 diabetes: A proof-of-concept study
Source: Front Clin Diabetes Healthc. 2022 Sep 23;3:986672. doi: 10.3389/fcdhc.2022.986672 (PMC10012122; doi:10.3389/fcdhc.2022.986672)
Supplement: Supplementary file 1 [file Table_1.docx]

**Online-Only Supplemental Material GlucoTab@MobileCare**

Supplemental Material

Table 1: Results from nurses complete questionnaire before study start (1= strongly disagree; 6 = strongly agree)

Table 2: Results from nurses complete questionnaire after study end (1= strongly disagree; 6 = strongly agree)

Table 3: Results from participants/relatives complete questionnaire at study end (1= strongly disagree; 6 = strongly agree)

Table 1: Results from nurses complete questionnaire before study start (1= strongly disagree; 6 = strongly agree)

Table 2: Results from nurses complete questionnaire after study end (1= strongly disagree; 6 = strongly agree)

Table 3: Results from participants/relatives complete questionnaire at study end (1= strongly disagree; 6 = strongly agree)
